# Psychosocial factors influencing smoking relapse among youth experiencing homelessness: A qualitative study

**DOI:** 10.1371/journal.pone.0270665

**Published:** 2022-07-26

**Authors:** Joanne G. Patterson, Joseph M. Macisco, Allison M. Glasser, Amy Wermert, Julianna M. Nemeth

**Affiliations:** 1 The Ohio State University Comprehensive Cancer Center, Columbus, Ohio, United States of America; 2 Division of Epidemiology, The Ohio State University College of Public Health, Columbus, Ohio, United States of America; 3 Division of Health Behavior and Health Promotion, The Ohio State University College of Public Health, Columbus, Ohio, United States of America; Rutland Regional Medical Center, UNITED STATES

## Abstract

**Objectives:**

In the United States, up to 70% of youth experiencing homelessness smoke cigarettes. Many are interested in quitting; however, little is known about psychosocial factors influencing smoking relapse in this population. This study, part of a larger project to develop an optimized smoking cessation intervention for youth experiencing homelessness, aimed to describe how psychosocial factors influence smoking relapse in this group.

**Methods:**

This study describes the smoking relapse experiences of 26 youth tobacco users, aged 14–24 years, who were recruited from a homeless drop-in center in Ohio. We conducted semi-structured interviews to understand how stress, opportunity, and coping contribute to smoking relapse.

**Results:**

Five themes emerged from the data: (1) smoking as a lapse in emotional self-regulation in response to stress; (2) smoking as active emotional self-regulation in response to stress; (3) social opportunities facilitate smoking in the context of emotion-focused stress coping; (4) problem-focused stress coping; and (5) opportunity facilitates smoking relapse.

**Conclusions:**

Stress was a primary driver of smoking relapse among youth experiencing homelessness, yet social and environmental opportunities to smoke also precipitated relapse. Interventions to improve abstinence among this population should target foundational stressors, coping skills, social supports, and nicotine dependence.

## Introduction

Tobacco use is a critical concern for youth experiencing homelessness. In the United States (US), an estimated 4.2 million youth aged 13–25 years experience unaccompanied homelessness annually [1]. These young people—including 1 in 10 youth aged 18–25 years and 1 in 30 minors aged 13–17 years [1]—endure sleeping on the streets, in shelters, or temporarily sleeping in others’ homes while unaccompanied by a parent or guardian. Alarmingly, up to 70% of youth experiencing homelessness report smoking cigarettes [2, 3], and many are poly-tobacco product users [4, 5]. In one study conducted in Los Angeles County, 72% of youth experiencing homelessness smokers reported alternative tobacco product use; including e-cigarettes (51%), little cigars/cigarillos (46%), hookah (31%), chewing tobacco (19%), and other smokeless tobacco products (e.g., Camel Snus, Sticks, or Orbs; 24%) [5]. Unsurprisingly, many youth experiencing homelessness also report daily smoking and exhibit strong nicotine dependence [6, 7]. High-risk smoking practices including sniping (i.e., smoking discarded cigarette butts) [7] and sharing cigarettes [6] are also commonly reported by this group.

Despite the high smoking prevalence among this population, youth experiencing homelessness are motivated to quit [5, 6, 8–10]. Across multiple studies, 32.5–43.4% of youth experiencing homelessness express an interest or motivation to quit cigarettes in the next 30 days [6, 8, 10]. Among youth experiencing homelessness who use alternative combustible tobacco products, 18.8–42.3% are motivated to quit [5]. Accordingly, a majority of youth experiencing homelessness report multiple quit attempts annually [6]. Among homeless young adults (aged 18–25 year) in Los Angeles County, 75% reported ever stopping smoking for at least 24 hours with the intent to quit [8]. On average, participants reported engaging in 1.9 quit attempts annually. In a study of youth experiencing homelessness (aged 13–25 years), fewer participants (65.7%) reported quitting cigarettes for at least 24 hours during the past year; however, they also reported a greater average number of past-year quit attempts (n = 9.2) [6]. Similarly, 32.3–52.3% of youth experiencing homelessness (aged 13–25 years) who used alternative combustible tobacco products reported one or more past year quit attempts [5]. These studies underscore that youth experiencing homelessness want to quit smoking and engage in multiple attempts.

A growing body of literature documents quit experiences among youth experiencing homelessness [4–6, 8, 10–12]. Most studies have focused on quit methods [5, 6, 8, 10, 11]; finding that youth experiencing homelessness are most likely to engage in unassisted quitting (i.e., without use of evidenced-based counseling, nicotine replacement therapy (NRT), or pharmacotherapy) [6, 11]. In contrast, youth experiencing homelessness rely on self-motivation and willpower to quit; often quitting “cold turkey” [6, 8, 11]. Youth also report substituting tobacco products (e.g., e-cigarettes, cigarillos) [5, 10, 11], other product substitution (i.e., marijuana) [11], rate reduction [8, 11], and behavioral distractions (e.g., exercise, art, eating candy) [8, 11] to support unassisted quit attempts. However, these strategies tend to produce only short-term quit success [11].

It is clear that youth experiencing homelessness are attempting to quit smoking with limited success. To our knowledge, no studies have documented the psychosocial factors precipitating smoking relapse in youth experiencing homelessness. This is a substantial gap in the literature, as understanding the factors that contribute to smoking relapse is critical for developing efficacious smoking cessation interventions for this population. In the sole study reporting barriers to quitting smoking among youth experiencing homelessness, young adults aged 18–25 cited stress and social influences (i.e., peer use) as primary barriers to quitting [8]. However, this study did not investigate factors precipitating smoking relapse after a quit attempt.

More studies have investigated barriers to smoking cessation among homeless adults [13–16], who also describe stress as a primary barrier to quitting [13, 14]. In a study of homeless adults living in shelters, residents specifically described major life events (e.g., birth) and daily hassles (i.e., work-related stress) as primary reasons for smoking initiation and relapse [15]. Similarly, in an earlier study, homeless adults described smoking as a primary means to cope with chronic strains associated with homelessness (e.g., poverty, lack of housing, hunger) and mental illness [16]. Participants also reported that smoking behaviors were influenced by opportunity; including high social acceptability of smoking and environments that enabled access to cigarettes [16]. It is likely that these same psychosocial factors (I.e., stress, opportunity, coping) influence smoking relapse in youth experiencing homelessness; however, no studies have investigated this question.

**Fig 1:**
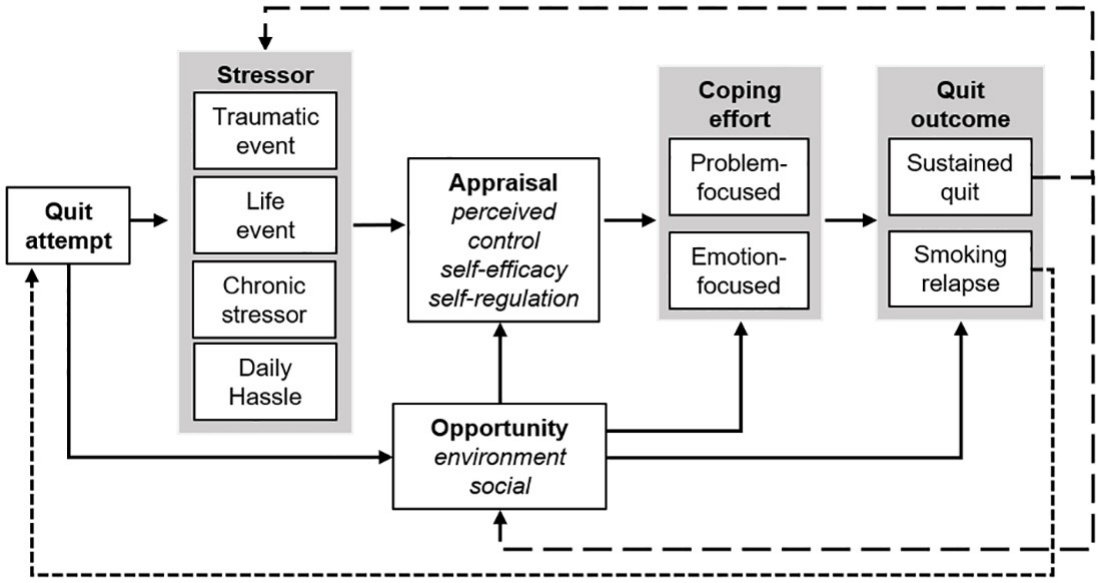
Simplified model of psychosocial factors predicting smoking relapse in youth experiencing homelessness.

Based on the scientific literature [8, 14–16] and extant theory [17, 18], we hypothesize that stress, opportunity, and coping may predict smoking relapse in youth experiencing homelessness (Fig 1).

The premise of this model is that youth experiencing homelessness are confronted with multiple types of stressors on a daily basis; including traumatic events (e.g., violence, deaths), life events (e.g., births, graduations), chronic stressors (e.g., poverty, hunger), and daily life hassles (e.g., arguments, travel difficulties). However, youth are also presented with opportunities to smoke (i.e., environment and social supports) to which they must respond. During a quit attempt, how youth respond to stressors and opportunities is based on their appraisal (conscious or unconscious) of the stressor or opportunity. These appraisals are informed by the youth’s perceived control, self-efficacy, and self-regulatory capacity at the time of the appraisal [17]. When faced with stressors, youth may respond with problem-focused coping (i.e., coping strategy directly addressing the stressor) or emotion-focused comping (i.e., coping strategy addresses the emotions arising from the stressor). Similarly, when faced with opportunities conducive to smoking (e.g., free or found cigarettes, peers who smoke), youth experiencing homelessness may also respond with problem- or emotion-focused coping efforts. How youth respond to stress and opportunity results in continued smoking abstinence or relapse.

The present study is part of a larger project designed to investigate the experiences of youth experiencing homelessness who use combustible tobacco, including their past quit attempts. The overall study goal is to develop an optimized smoking cessation intervention for youth experiencing homelessness. Given the dearth of information on factors precipitating smoking relapse in this population, our study applies a theoretically- and empirically-grounded model to guide our qualitative inquiry into the psychosocial processes influencing smoking relapse in youth experiencing homelessness aged 14–24 years old. Specifically, our study aimed to describe how stress, opportunity, and coping influence smoking relapse in youth experiencing homelessness after a quit attempt.

## Methods

### Study design

In 2019, study personnel conducted 30–60 minute, in-person, qualitative interviews at a drop-in center for youth experiencing homelessness, with past-week combustible tobacco users. The study design has been described elsewhere [12]. We obtained written informed consent for participants aged 18–24 years and written assent for participants aged 14–17 years. To minimize risk for young participants experiencing difficult parental relationships (e.g., family history of violence, neglect), we obtained a waiver for parental consent for youth aged 14–17 years. Interview participants received a $25 gift card to a local grocery store. The study was approved by The Ohio State University’s Institutional Review Board (Protocol #2017C0148).

### Participants

Participants included youth experiencing homelessness attending a drop-in center in a Midwestern city. Eligibility required that participants be (1) aged 14 to 24 years, (2) current combustible tobacco smokers (defined as smoking some or every day in the past seven days), and (3) meet 2002 McKinney-Vento Act criteria for homelessness (i.e., lacking fixed, regular, and adequate nighttime residence; living in a welfare hotel or place without regular sleeping accommodations; or living in a shared residence with other persons due to loss of housing or economic hardship) [19]. This study is a sub-analysis of a larger project; for this study, participants had to describe smoking relapse. That is, they responded to the following interview questions: “What happened right before you started smoking again?” and “What do you think caused you to relapse?” A total of 36 participants were recruited into the broader study. Data collection and analysis was iterative. Participant recruitment concluded when thematic saturation was achieved such that no new themes were elicited as we sampled additional participants [20]. Of all youth, 33 (91.7%) reported a past quit attempt. Twenty-six participants (72% of full sample) reported a reason for smoking relapse and were included in this analysis.

### Study instruments

We used a semi-structured interview guide for in-person interviews. Grounded in the Capabilities-Opportunity-Motivation for Behavior (COM-B) model [21], a theoretical framework that explains the psychosocial and environmental factors underlying behavior, interview questions elicited data on the environmental and psychosocial factors influencing participants’ smoking behaviors, including current smoking, past quit attempts, and engagement in future supported cessation. Following the interview, participants completed a demographic questionnaire.

### Data analysis

Audio-recorded interviews were transcribed by a professional third-party team and uploaded into ATLAS.Ti 8.4.2 (Atlas.ti Scientific Software Development GmbH, Berlin, Germany) for data management and analysis. Using a team-based approach, study personnel first classified interview data (i.e., text) into thought units (i.e., a phrase, sentence, or paragraph that conveys a single meaning). Thought units were reviewed by the study team until agreement was reached. For this study, only thought units that emerged in response to questions concerning reasons for smoking relapse were coded.

Data analysis incorporated a deductive template approach using thematically-grounded a priori codes [22] and a data-driven inductive approach [23]. Study personnel (JMM, JGP) applied concepts from stress and coping theory [17, 18] (Fig 1) to develop *a priori*, theoretically-informed codes. Deductive code groups included: reason for relapse (stress, opportunity, other), stressor type (trauma, life event, chronic stressors, daily hassles), opportunity (social or environmental), appraisal (self-regulation), and coping strategy (emotion- or problem-focused).

After reading all relevant data repeatedly to achieve immersion, we applied deductive codes to all data (JMM, JGP). We also coded data inductively, allowing unique themes to emerge from the data. Inductive codes included quit methods, nicotine dependence, and social supports. The final codebook described four deductive and four inductive codes, including a definition for each code and example quotations from the data (Table 1). The research team met weekly to discuss coding procedures and progress. A total of 133 codes were assigned across 79 thought units (mean = 3.0 thought units per participant). An initial coding comparison indicated moderate percent agreement (83%) for 8 code groups across 133 thought units comprising the 26 source interviews. Coding discrepancies were discussed until agreement was reached. A third researcher (JMN) reviewed data to ensure that selected text (data) appropriately represented codes and that codes were appropriately assigned to code groups.

**Table 1:** Sample codebook.

Code	Definition
Stress	Participant reports something stressful that causes them to smoke again
Opportunity	Participant reports returning to smoking because the opportunity presented itself
Other	Participant reports relapse was precipitated by a factor that was *neither* stress nor opportunity.
Trauma	Participant reports experiencing a traumatic event (trauma: threat of physical integrity, injury, or death to self or others, the person experiences fear, helplessness, or horror and results in stress))
Life events	Participant reports big changes in short period of time (e.g., death, first baby, divorce)
Chronic strains	Participant reports relatively smaller changes over long periods of time (e.g., disabling injury, poverty)
Daily life hassles	Participant reports mini-events that require change during a day (e.g., traffic jam, unexpected guest, bad day at work)
Self-regulation	Participant has a lapse in controlling one’s emotions, thoughts, or behaviors OR uses tobacco as a way to restore their emotions, thoughts, and behaviors from a disrupted state)
Problem-focused coping	Participant identifies or uses a coping mechanism to manage the stressor itself)
Emotion-focused coping	Participant identifies or uses a coping mechanism to manage the emotions associated with the stressor)
Quit methods	Participant describes method used during quit attempt
Nicotine dependence	Participant describes physiological or psychological symptoms of nicotine dependence (e.g., cravings, withdrawal, irritability, mood swings, headache).
Social supports	Participant describes their social support system in the context of a quit attempt and relapse

Five themes emerged during the coding process: (1) smoking as a lapse in emotional self-regulation in response to stress; (2) smoking as active emotional self-regulation in response to stress; (3) social opportunities facilitate smoking in the context of emotion-focused stress coping; (4) problem-focused stress coping; and (5) opportunity facilitates smoking relapse. Specific coding groups (i.e., nicotine dependence, social supports) cut across all themes and so are integrated within the narrative where appropriate.

## Results

### Sample characteristics

Twenty-six participants responded to smoking relapse questions during interview (Table 2); 65.4% of the sample was aged 18–24. The sample mostly self-identified as female (50.0%) and 57.7% self-reported their race/ethnicity as Black, African American, or African National. Most (76.9%) were current daily smokers and reported being willing to quit in the next 30 days (69.2%).

**Table 2:** Sample characteristics of youth experiencing homelessness who reported a smoking quit attempt and relapsed (N = 26).

	Full Sample	Age 14–17 years	Age 18–24 years
	n (%)	n (%)	
Total N	26 (100)	9 (34.6)	17 (65.4)
Gender			
Female	13 (50.0)	7 (77.8)	6 (35.3)
Male	11 (42.3)	2 (22.2)	9 (52.9)
Gender Non-conforming	2 (7.7)	0 (0.0)	2 (11.8)
Race/ethnicity			
Black, African American, or African National	15 (57.7)	6 (66.7)	9 (52.9)
Native American	1 (3.9)	0 (0.0)	1 (5.9)
Non-Hispanic White	5 (19.2)	1 (11.1)	4 (23.5)
Other	5 (19.2)	2 (22.2)	3 (17.6)
Current daily smoker	20 (76.9)	8 (88.9)	12 (70.6)
Willing to quit smoking, next 30 days	18 (69.2)	7 (77.8)	11 (64.7)
Reason left family of origin^1^			
Physical, sexual, or verbal abuse	6 (23.1)	2 (22.2)	4 (23.5)
Own substance abuse	1 (3.8)	0 (0.0)	1 (5.9)
Thrown out	12 (46.2)	5 (55.6)	7 (41.2)
Arguments with parents/family	8 (30.8)	4 (44.4)	4 (23.5)
Death of parents/family	2 (7.7)	1 (11.1)	1 (5.9)
Other	8 (30.8)	2 (22.2)	6 (35.3)
Spent night(s) during the past 30 days^1^			
With family members		5 (55.6)	4 (23.5)
With an intimate partner		2 (22.2)	1 (5.9)
With friends in their home		4 (44.4)	4 (23.5)
In a shelter or mission		1 (11.1)	6 (35.3)
In a squat or abandoned building		0 (0.0)	1 (5.9)
Outdoors on the street, or in a park or alley		2 (22.2)	0 (0.0)
Outdoors in a bus or train station or airport		1 (11.1)	4 (23.5)
In a residential treatment program		0 (0.0)	2 (11.8)
Other		2 (22.2)	2 (11.8)

^1^ As participants could choose more than one response category, percentages across categories may sum to more than 100%.

Of the 26 youth reporting smoking relapse, 23 (88.5%) discussed stress or opportunity as reasons for relapse. Fifteen participants (65.2%) reported stress as their sole reason for relapse while three participants (13.0%) reported opportunity as their sole reason for relapse. An additional five participants (21.7%) reported that it was the intersection of stress and opportunity that precipitated smoking relapse.

### Theme 1: Smoking as a lapse in emotional self-regulation in response to stress

Youth most often reported experiencing major life events prior to relapse (n = 9, 39.1%). These events were traumatic and ranged from the forced removal of children into the foster care system, death of friends and family due to debilitating chronic diseases (e.g., cancer) or accidental drug overdose, and physical violence. When faced with trauma, youth experiencing homelessness often described feeling emotionally shaken or “broken”. One female participant noted, “I lost my friend… she overdosed in front of me. I was there the whole night and I didn’t know, so that broke my insides again. I couldn’t sleep, all I wanted to do to keep myself up was to smoke and just think about it, I was in shock” (Female, aged 14–17 years). After experiencing trauma, youth experiencing homelessness were overwhelmed by negative feelings, which then prompted smoking relapse. Smoking was presented as a reflexive action, or lapse in emotional self-regulation. For example, after her brother was violently assaulted, a female participant described self-medicating with cigarettes while faced with exhaustive and unrelenting emotions: “So, while he was at the hospital, I just went downstairs and smoked a cigarette cuz I couldn’t stop crying the whole entire day” (Female, aged 14–17 years).

Youth also described how nicotine dependence intersected with emotion-focused coping in response to stressful events. In addition to describing a lapse in their ability to self-regulate emotions, these participants described physiological cravings; a “need” or “urge” to smoke. As said one female participant, “It was more like a desperate thing, I needed something to smoke. I was really irritated and mad and things was not going right. So, it was like “I don’t care no more”, and I just hit that first cigarette” (Female, aged 14–17 years). For this young woman, nicotine addiction overwhelmed her ability to cope with stress while remaining abstinent, which precipitated smoking relapse.

For other youth, experiencing the physiological symptoms of nicotine dependence (i.e., withdrawal) was the stressful event that precipitated relapse.

INTERVIEWER: Okay, and you said you went like just a couple of days without smoking.
PARTICIPANT: In a couple of days, and I went nope, I give up.
INTERVIEWER: So what happened right before you started smoking again?
PARTICIPANT: I felt nauseous. I was like, man, maybe I shouldn’t smoke this, but I went ahead and smoked it. And I coughed like it wasn’t anything. It made me feel high as well….I just think it was my body is telling me I needed a cigarette.

Others described how physiological nicotine dependence, withdrawal, and stress were circularly entwined:

Participant: I’ll have bad anxiety, so I’ll just lit that cigarette up….
Interviewer: What kind of happened right before you started smoking again?
Participant: Lightheaded, light headed, shaking. My God, damn nicotine all in my body, for real. Man, for real, serious. Man, I’m kind of stressed. I feel kinda better. That’s all I kept thinking. I think cuz people smoke cigarettes when they just stressed, so yeah, I felt way better a little bit, and, just that’s big. (Male, aged 18–24 years)

Initially, the participant described smoking to manage stress (here, characterized by anxiety). When trying to quit, he experienced nicotine withdrawal, which further physiologically stressed his body. To reduce the stress of withdrawal he smoked a cigarette; ultimately, reinforcing a pattern of reflexive smoking and emotion-focused stress coping.

Finally, while in the minority, a few participants described trauma and smoking relapse in objective tones that belied the magnitude of the stressor being described.

My mom died. So, I found myself a nice lovely cigarette at the corner store. And I smoked it. And then I ended up buying a pack, and then next thing I know it went downhill from there.

(Male, aged 18–24 years)

In this case, smoking was presented as an automatic response apropos to experiencing traumatic life events. Contextually, most participants described experiencing multiple traumatic events over the life course. As such, it is possible that such “matter of fact” presentations of smoking and relapse reflect avoidant emotion-focused coping (i.e., numbing) after experiencing multiple or repeated traumas.

### Theme 2: Smoking as active emotional self-regulation in response to stress

Participants also described how they actively chose to smoke to self-regulate stress-induced emotions. In these situations, participants engaged in a cognitive process that weighed the costs and benefits of varied emotion-focused coping strategies, including smoking. One young mother described how she returned to smoking after the compulsory removal of her two sons into foster care.

I was fighting for my kids… And it became more stressful. And it was like I didn’t want to go out and do anything stupid. And I felt like since smoking was a way for me to relieve my stress before, hey, why not smoke a cigarette?

(Female, 18–24 y/o)

In the face of limited choices, smoking was the safest stress-coping strategy for this young mother. In this scenario, smoking was presented as a harm reduction strategy, in which the participant chose smoking to avoid engaging in other risky behaviors (e.g., alcohol or illicit drug use, physical altercations) that could compromise her chances of regaining child custody.

Youth experiencing homelessness also described using smoking as a harm reduction strategy to cope with stress arising from daily life hassles, including disagreements with peers and family members. These participants described choosing to smoke in lieu of physically hurting their loved ones. As shared one male participant, “… Well, you can’t really put your hands on her [his girlfriend]. You can’t really yell at her, it doesn’t really work. So, smoke a cigarette” (Male, aged 18–24 years). This same decision—to avoid physical violence via smoking—was echoed by a young female participant: “Me and my brother we got into an argument. And I just got so mad I left and I went to go get me a port [Newport]…Instead of going out and hurting, I just took my anger out on that cigarette, just relieved from there” (Female, aged 14–17 years). For this participant, anger was not merely diffused, but keenly redirected into the act of smoking, which eventually brought stress-relief.

### Theme 3: Social opportunity facilitates smoking in the context of emotion-focused stress coping

While describing stressful events, participants revealed how social opportunities facilitated smoking relapse in the context of emotion-focused stress coping. For one young man, smoking relapse was precipitated by job-related stress.

Participant: Something at work pissed me off. And I was working my ass off and people were messing, I don’t know. I don’t even know what I was mad about, but I know it was about the job. I’m not, fuck, I need a cigarette. (Male, aged 18–24 years)

Here the participant described how he reflexively craved a cigarette to cope with his anger in response to work-related stress. Work featured in multiple youths’ lives as an important mechanism for securing basic needs, including food and housing. Consequently, it is unsurprising that work hassles—and especially those that might affect performance and job security—were stressful for participants. However, this young man’s smoking relapse was also facilitated by opportunity:

Interviewer: Who provided you that cigarette?
Participant: My boss…

Having decided to quit smoking, this participant did not have access to cigarettes while on shift at work. However, his boss provided him a cigarette.

Other participants also described how their social circles provided them an opportunity to access tobacco during quit attempts. As one young female participant described, “…they just throw out the cigarettes, and I was just, “My God. I don’t got nothing to smoke. Give me one of those”” (Female, aged 14–17 years). In the face of stress-induced emotions, social smoking opportunities were powerful “in-the-moment” facilitators of relapse.

### Theme 4: Problem-focused stress coping

Only one individual reported using problem-focused coping to manage stress. After finding out she was pregnant, her father kicked her out of their home. Facing homelessness, she looked for resources and found them in a drop-in center.

I was in school. And this is actually how I found out about [the drop-in center]… So it was, that triggered my stress a lot. Because then I felt like nobody was here for me…My dad don’t love me anymore; I’m pregnant now. I’m in this big city I barely know anything about. I just got here a year ago, so I don’t know anything and now I gotta live. And live for this baby that’s growing inside of me, and make a way, and still get out of that. So yeah, it was pretty stressful, but I got out of it. The [youth drop-in center] was definitely a help. They definitely helped a lot. When I was pregnant”

(Female, aged 18–24 years).

Yet, despite leveraging problem-focused coping strategies, she relapses after a 2-year long abstinence from smoking. “So, it was something that triggered that stress that make me wanna start smoking cigarettes, and the crowd that I was hanging around too” (Female, 18–24 y/o). Ultimately, permissive smoking norms, social access to tobacco, and the stress of pregnancy and homelessness overwhelmed her problem-focused coping efforts.

### Theme 5: Opportunity facilitates smoking relapse

Only four participants (17.4%) reported opportunity as their primary reason for relapse. For these individuals, peer networks facilitated access to cigarettes during periods of smoking abstinence. When describing these incidents, two participants used language indicating dependence on nicotine. One young woman described, “…the people I hung around with and my boyfriend and stuff was always smoking. Just couldn’t resist it” (Female, aged 18–24 years). Here the description is vague—she couldn’t “resist” smoking. However, a second participant describes this process in more detail:

And when I see that cigarette, I was like, I can’t do it. I felt nauseous. I was like, “Man, maybe I shouldn’t smoke this”, but I went ahead and smoked it. And I coughed like it wasn’t anything. It made me feel high as well… I just think it was my body is telling me I needed a cigarette.

(Female, aged 18–24 years)

Here the participant’s decision to smoke was precipitated by opportunity—social access to cigarettes in her peer group. However, it was also influenced by nicotine dependence as characterized by physical nausea and a belief that her body “needed a cigarette”. In such situations, opportunity and nicotine dependence overwhelmed youths’ capacity to sustain smoking abstinence.

Only one participant described how his physical environment precipitated smoking relapse. This participant had quit smoking by substituting vaping for cigarettes. He described returning to the tobacco store where he previously purchased vaping supplies:

I went to go get another vape pen and the same manufacturer guy was there, but he gave me a crazy deal on cigarettes. I got American Spirits for $8 a piece, and for 4 bucks, he gave me 2 packs. So, I got my girlfriend to get a coupon too, and then my friend got a coupon. So we ended up getting six packs for ten bucks.

(Male, aged 18–24 years)

Exposure to “crazy” cigarette price promotions plus permissive social tobacco norms (i.e., friend and girlfriend who facilitated purchase) facilitated this participant’s return to smoking.

## Discussion

The goal of this qualitative study was to describe how stress, opportunity, and coping influence smoking relapse in youth experiencing homelessness. Consonant with the literature documenting the experiences of homeless adults [15], youth experiencing homelessness described how traumatic and major life stressors (e.g., poverty, unstable housing, peer/familial conflict) directly preceded smoking relapse. That youth experiencing homelessness experience substantial trauma is not novel [24]; however, our results suggest that smoking cessation interventions must support youth experiencing homelessness during a critical window of vulnerability—when they are faced with myriad stressors that prompt them to return to tobacco in-the-moment.

Youth experiencing homelessness in our study reported smoking to self-medicate negative emotions, boost mood, and manage anger. While smoking to self-regulate stress is a universal theme among youth regardless of housing status [25], this experience is complicated for youth experiencing homelessness who are more likely to experience serious mental health disorders (e.g., depression, post-traumatic stress disorder) [26]. Between 35–76% of youth experiencing homelessness also meet diagnostic criteria for multiple diagnoses (e.g., comorbid substance use and serious mental health disorders) [27], which diminish their capacity to self-regulate emotions. Our findings suggest that supportive cessation interventions for youth experiencing homelessness must be trauma-informed and attend to the emotional, cognitive, and behavioral effects of trauma [24, 28]. Limited research has been conducted on trauma-informed smoking cessation interventions, but studies suggest that using trauma-informed approaches (e.g., using non-judgmental language, prioritizing emotional and physical safety) benefits vulnerable populations [29, 30].

Ultimately, increasing smoking abstinence for youth experiencing homelessness requires addressing the foundational stressors that precipitate relapse, including poverty, safe housing and job instability, restricted access to goods and services, and more. As reported by participants in this study and others [8] coping with the daily and accumulated stress of living with unmet foundational needs is a substantial reason for smoking (or returning to smoking). Similarly, Bagget and colleagues found that homeless adults who had less access to foundational needs (e.g. food, shelter, clothing) also had greater perceived barriers to quitting smoking and a reduced odds of remaining abstinent when compared to homeless adults who had greater access to foundational needs [31]. Helping youth experiencing homelessness meet their foundational needs is an opportunity for intervention that could also support smoking abstinence among youth experiencing homelessness. However, future studies are needed to test this hypothesis.

Smoking cessation programs targeting this population must also account for the specific cognitive, emotional, and social factors that precipitate smoking relapse. Youth in our sample predominantly described smoking relapse as a reflexive action in which they impulsively smoked to reduce overwhelming or prolonged negative emotions. Among youth in general, impulsivity is associated with cigarette craving [32] and use [33] among regular smokers, and adolescents who are more impulsive (i.e., those who make decisions without future thinking) are less likely to maintain smoking abstinence [34, 35]. It is important to note that impulsivity, especially during adolescence, can be influenced by a myriad of factors, including normal development of the adolescent brain combined with limited experience engaging in novel behaviors, mental health conditions and other substance use, and acting without thinking due to executive function dysregulation (e.g., in response to traumatic brain injury) [36]. For, youth experiencing homelessness, exposure to high rates of adverse environmental experiences (e.g., physical violence [37]) may exacerbate executive function dysregulation due to brain injury [38] or the physiological effects of trauma [39, 40]. Addressing impulsivity in this population thus requires identifying the source of impulsivity and intervening to accommodate (in the case of injury) or co-treat (in case of injury or co-morbid substance use/mental health). It is also likely that interventions may need to attend to environmental factors contributing to sources of impulsivity by addressing the contexts in which youth are making cessation attempts (e.g., by reducing exposure to violence or other social or structural determinants of health).

To maintain abstinence, targeted smoking cessation interventions may require components that bolster youths’ capacity to redirect impulsive behavior without a return to tobacco. Promising interventions leverage ecological momentary assessments (EMA) collected via mobile phone to predict imminent smoking relapse in low income adults [41] and young adults experiencing homelessness [42] by delivering just-in-time cessation counseling tailored to address specific relapse triggers (e.g., stress). Specifically, Tucker and colleagues (2021) pilot-tested a text messaging-based intervention, as an adjunct to brief smoking cessation counseling plus NRT (i.e., standard treatment), among young adults experiencing homelessness. Compared to the standard treatment condition, participants in the text messaging condition reported greater 7-day point prevalence abstinence at 90-day follow-up and greater reductions in the number of days smoked in the past 30 days [42]. These results echo early EMA studies with low income adults that demonstrate preliminary efficacy for reducing participants’ self-reported urges to smoke [43] and maintaining smoking abstinence [44]. Given these early results, larger studies testing the effectiveness of text-based, EMA-informed interventions to support youth experiencing homelessness during quit attempts are warranted.

At times, youth were more aware of their stress-coping behaviors. In these cases, while youth experienced strong negative emotions in response to stress, they described deliberately choosing to smoke for stress-reduction. In addition to calming heightened emotions, participants claimed that smoking relapse helped them avoid more detrimental behaviors (e.g., physical violence). During stressful and traumatic events, the mathematics of smoking relapse was simple: a return to smoking produced short-term gains in emotion regulation and harm reduction. As such, smoking cessation interventions may need to incorporate cognitive-behavioral strategies to help youth identify and apply alternate stress-reducing tools [45]. Mindfulness-based practices show promise for enhancing smoking cessation via response inhibition, improved mood/affect, and emotion regulation [46–49]. However, more research is needed to understand the impact of mindfulness-based practices on smoking cessation, including the mechanisms through which these approaches may be effective, and for whom.

Youth experiencing homelessness also described examples where opportunity (i.e., social supports and environmental cues) precipitated relapse. That is, when youth experienced a stressful event during a period of smoking abstinence, social supports that provided an opportunity to smoke that increased the likelihood of engaging in emotion-focused coping, leading to relapse. Consistent with the literature among youth experiencing homeless [8, 50] and in the general population [25], peer networks strongly contributed to cessation relapse by providing access to cigarettes and encouragement to smoke. For youth experiencing homelessness, this behavior may be reinforced by the broad availability of smoking spaces in shelter and day services programs [50]. In the general population, school-based smoking interventions have utilized motivational interviewing and group-based lessons to increase smoking refusal self-efficacy among students, and social support for quitting has been shown to improve smoking cessation intervention effectiveness [51, 52]. Future research is needed to assess the feasibility and acceptability of these intervention components for youth experiencing homelessness in the context of drop-in programs.

Finally, most youth experiencing homelessness in our sample reported daily smoking; unsurprisingly, participants also described how nicotine withdrawal precipitated relapse post-quit attempt. To support nicotine dependent youth during quit attempts, cessation interventions for youth experiencing homelessness may be strengthened by providing pharmacotherapy alongside behavioral treatments. Pharmacotherapy options include NRT (e.g., nicotine patches, gum) and medication (i.e., bupropion, varenicline). In the most recent Clinical Practice Guidelines [53], pharmacotherapy was not recommended for adolescents given lacking experimental evidence. However, in a recent meta-analysis of youth smoking cessation trials, pharmacotherapy (vs. control) was associated with increased smoking abstinence at follow-up [54]. Early studies suggest that NRT is a feasible treatment option for youth experiencing homelessness. In a pilot evaluation of cessation counseling plus NRT with young adults experiencing homelessness, approximately 75% of participants reporting using provided nicotine patches [42]. On average, participants used 7–10 patches over a 30-day follow-up period; equivalent to 7–10 days of patch use [42]. In our prior work, about half of youth experiencing homelessness who attempted to quit tried NRT, but none used medication [11]. Youth experienced physical side effects (e.g., nausea) from NRT, yet most agreed that NRT should be an option in monitored smoking cessation programs for youth experiencing homelessness [11]. Youth were less enthusiastic about medication and expressed concerns about side effects and abuse liability [11]. In a qualitative study with service providers, providing pharmacotherapy to youth experiencing homelessness was viewed as critical for mitigating nicotine dependence during quit attempts [55]. Given evidence supporting the feasibility of NRT use among youth experiencing homelessness, studies testing strategies to increase and sustain NRT use among this population during quit attempts are warranted. However, research is also needed to evaluate the feasibility, acceptability, and preliminary efficacy of prescribing medication for smoking cessation in this high-risk group.

### Strengths and limitations

This study provides an important perspective on the behavioral and environmental factors precipitating smoking relapse among youth experiencing homelessness; however, it is not without limitations. As formative research, this qualitative study has produced some of the first knowledge of factors precipitating smoking relapse in a highly vulnerable and underserved population, yet our sample size was geographically-bound and is not generalizable to U.S. population of youth experiencing homelessness. Future multi-site studies are warranted to ascertain place-based differences in stressors associated with homelessness (e.g., poverty, unstable housing) and resources available to youth experiencing homelessness that may influence stress-coping and cessation lapse. Prior studies have determined associations between mental health, substance use, and smoking among young adults in the general population [56] and adults experiencing homelessness [57]; however, our study did not include focused questions about the role of these factors in smoking cessation and relapse. Future studies should assess the role of comorbid mental health and substance use issues on tobacco use, cessation, and relapse in this population. As with all descriptive human subjects research, there is a risk for social desirability bias. This concern is especially salient for youth experiencing homelessness who have limited resources and could be unduly influenced by financial incentives. However, we believe our $25 incentive, distributed as a grocery store gift card, was appropriate compensation for interview participation.

## Conclusion

For youth experiencing homelessness, stress coping is a primary driver of smoking relapse after a quit attempt; however, social and environmental opportunities to smoke also precipitated relapse. Our findings suggest that behavioral smoking cessation interventions for youth experiencing homelessness must employ a trauma-informed perspective; incorporate cognitive-behavioral strategies that target stress coping, impulsivity, tobacco refusal skills; and offer pharmacotherapy for nicotine dependence.
